# Cytotoxic T lymphocytes from cattle sharing the same MHC class I haplotype and immunized with live *Theileria parva* sporozoites differ in antigenic specificity

**DOI:** 10.1186/s13104-018-3145-8

**Published:** 2018-01-17

**Authors:** Lucilla Steinaa, Nicholas Svitek, Elias Awino, Rosemary Saya, Philip Toye

**Affiliations:** International Livestock Research Institute, P.O. Box 30709, Nairobi, 00100 Kenya

**Keywords:** *Theileria parva*, Cytotoxic T cells, Immunity, Strain specificity, MHC restriction

## Abstract

**Objectives:**

The objective of this study was to assess whether cytotoxic T cells (CTL) generated by the live vaccine, known as “ITM Muguga cocktail”, which is used for the cattle disease East Cost fever (ECF) in Sub-Saharan Africa, showed a broad reactivity against many different strains of the causative parasite *Theileria parva*. We also assessed whether immune responses were similar in cattle expressing the same MHC class I haplotypes.

**Results:**

The antigenic specificity of CTL from MHC class I-matched cattle vaccinated with the Muguga cocktail were different. Three cattle of MHC class I haplotype A18, one A18/A19 and two haploidentical (A18v/A12) animals, showed differential recognition of autologous cells infected with a panel of *T. parva* isolates. This could have implications in the field where certain strains could break through the vaccine. Furthermore, neither of the haploidentical cattle recognized the CTL epitope (Tp1_214–224_), presented by the A18 haplotype, in contrast to the third animal, showing differences in immunodominance in animals of the same haplotype A18. This suggests that the CTL specificities following immunization with the Muguga cocktail can vary even between haploidentical individuals and that some parasite strains may break through immunity generated by the Muguga cocktail.

**Electronic supplementary material:**

The online version of this article (10.1186/s13104-018-3145-8) contains supplementary material, which is available to authorized users.

## Introduction

*Theileria parva* is a tick-borne protozoan parasite which causes an acute and usually fatal cattle disease, known as East Coast fever (ECF), one of the most important cattle diseases in eastern and central Africa. ECF can eradicate up to 70% of the herd and can therefore confer serious impact [1]. ECF has been listed by the Food and Agriculture Organization of the United Nations (FAO) and by the World Organization for Animal Health (OIE) as a high priority disease to control to improve livelihoods of poor smallholder farmers [2].

The parasite infects bovine lymphocytes which subsequently undergo blast transformation and rapid multiplication [3], which usually results in overwhelming parasitosis and death within 2–4 weeks of infection. Cattle which recover from natural infection develop strong immunity to subsequent challenge. This has been exploited in a vaccination procedure, the “Infection and Treatment Method” (ITM), where live sporozoites are administered simultaneously with oxytetracycline. The main protective mechanism in both vaccinated and naturally recovered animals is believed to be cytotoxic (CD8^+^) T lymphocyte (CTL) killing of infected lymphocytes [4, 5].

Strain specificity of the protective response induced by ITM was initially observed in vivo by Radley et al. [6], and Irvin et al. [7], by immunizing with one strain and challenging with another. However, Radley et al. (1975) showed that immunization with a mixture of stabilates from three parasite isolates (Muguga, Serengeti-transformed and Kiambu 5) induced a broader protection to heterologous challenge than immunization with single isolates [8]. The mixture, known as the “Muguga cocktail”, is the basis of a commercial ITM vaccine, which appears to provide broad protection against *T. parva* in the field [9, 10]. Strain specificity of the CTL response has also been described but mostly in context of CTL clones [5, 11–14].

Various CTL antigens have been identified some of which are polymorphic [15, 16]. One such epitope (Tp1_214–224_) from the Tp1 antigen is presented by the A18 haplotype [17] and varies among different strains, which can affect recognition of infected cells by some CTL clones [18]. This does not seem to be a general phenomenon and was not the case when polyclonal responses were evaluated [19].

In the present study we investigated the CTL response to immunization with the Muguga cocktail in three animals of the MHC class I (BoLA) A18 haplotype, two of which were haploidentical. We investigated the specificity of the CTL on a panel of different strains to elucidate the breadth of the response and determined if the immunized animals recognized two variants of the Tp1_214–224_ epitope, which has previously been reported in animals immunized with the Muguga stabilate.

## Main text

### Methods

#### Animals

Male cattle **(***Bos taurus*, Friesian) were bought from farms in the Nyeri area in Kenya, tested free of tickborne diseases and subsequently MHC-typed using MHC class-specific antibodies (ILRI antibodies B4/18 and ILA35), followed by determination of haplotype as previously described [20, 21]. Briefly, RNA was extracted from PBMC, used with primer pairs for A10, A11, A12, A14, A15, A17, A18, A19, A20 and A31 class I haplotypes (Additional file 1) in a RT-PCR assay. Products were analyzed on a 1% agarose gel, purified with the QIAquick PCR Purification Kit (QIAGEN, cat # 28104) and sequenced on an ABI 3730 DNA Analyzer using the same primers used for the PCR reaction. Following this, three A18+ animals were selected for this study. Two cattle (BE033 and BE043) expressed the A12 haplotype (haploidentical), while one (BE017) expressed the A19 haplotype. The haploidentical animals expressed the variant A18 allele (BoLA-6*01302), whereas BE017 expressed the canonical allele BoLA-6*01301. A fourth animal (BE029) of the A14/A33 haplotypes was included as a control animal. Cattle were kept in a standard pen and subjected to standard husbandry procedures.

#### Immunization

Cattle were immunized intramuscularly, using ITM, in front and below the right ear using the Muguga cocktail, batch ILRI080, diluted 1:20 and immediately treated with tetracycline [22]. Animals were boosted 5 weeks after immunization using the vaccine without oxytetracycline.

#### PBMC separation

PBMC were purified by standard density centrifugation using Ficoll-Paque [23].

#### Parasitized cell lines

Cell lines infected with *T. parva* were established by infection of PBMC with sporozoites, as described previously [24]. Briefly, PBMC were infected with sporozoites by adding crushed dissected salivary gland from infected ticks. Two weeks later, small immortalized colonies were visible, which could be expanded further. Cryopreserved sporozoites of the reference stabilates: Muguga 4230, Serengeti-transformed 4229, Kiambu 5 4228, components included in ILRI0801 [22], were used for establishment of cell lines. Five cloned stabilates were also used: Marikebuni 3292, buffalo-derived 3570, Boleni 3198, [25], Mariakani 3212 (unpublished; cloned from stabilate 1937) and Uganda 3645 (single passage from stabilate 3569) [25].

#### Peptides

Peptides representing the CTL epitope in Tp1 (purity > 95%, Mimotopes, Clayton, Australia) were used for pulsing of PBMC as target cells in CTL and ELISPOT assays. Tp1_214–224_ Muguga: VGYPKVKEEML; TP1_214–224_ Marikebuni: VGYPKVKEEII.

#### Generation of CTL

CTL bulk cultures were generated by stimulation of PBMC with a mixture of irradiated autologous cell lines, each infected with one of the three reference stabilates of the Muguga cocktail in a ratio of 10:1 (PBMC vs. stimulator cells). Three restimulations were performed before the CTL assay was made. For reactivity to the Tp1 epitope, CTL were expanded using autologous cells infected with the Muguga 4230 reference stabilate. The procedure was essentially as described before [23].

#### Tetramer analysis

Staining of PBMC with tetramer and CD8 antibody was essentially as described in Svitek et al. [23]. Twenty five microliters PE-labeled Tp1_214–224_ tetramer (provided by Pierre van der Bruggen, Ludwig Institute for Cancer, Brussels) was used to stain 4 × 10^5^ cells to a final concentration of 40 nM.

#### Cytotoxicity assay

Standard 4 h release assay using ^51^Cr-labeled target cells were used to measure cytotoxicity, essentially as described in Svitek et al. [23].

### Results

#### Strain specificity of CTL from immunized animals

Three animals of the A18 haplotype were selected for these experiments. One calf (BE017) expressed the canonical A18 allele (BoLA-6*01301), with the other haplotype being A19. The other two cattle (BE033 and BE043) expressed the variant A18 allele (BoLA-6*01302) and the other haplotype was determined to be A12, and so they are considered to be haploidentical. The two A18 alleles differ by a single amino acid, Glu → Leu at position 97 [22].

**Fig. 1:**
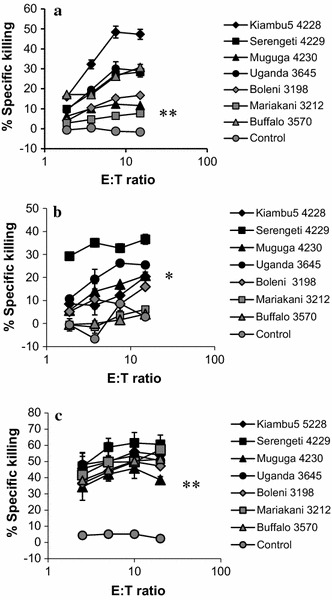
Strain-specific response by CTL generated in three A18+ animals. BE017 (**a**), BE033 (**b**) and BE043 (**c**). Seven infected cell lines were prepared from each animal as detailed in the Methods. The CTL lines from were tested at several effector:target (E:T) ratios as indicated. The experiment was performed twice with similar results. Significance compared to the PBMC control was tested with a T-test. *P < 0.05. **P < 0.01. **a** All P < 0.05; **b** buffalo and Mariakani were not significant different from PBMC, all other P < 0.05; **c** all P < 0.01

Initially, CTL lines from each of the three A18+ animals were tested on autologous cell lines infected with each of the three component stabilates of the Muguga cocktail or with one of four cloned stabilates: Boleni, Mariakani, Uganda and a buffalo-derived strain. CTL from the three cattle showed different patterns of cytotoxic activity on the target cell lines (Fig. 1). BE017 recognized all cell lines, although with differences in intensity. CTL from BE043 showed consistently strong killing of all cell lines, whereas CTL from the haploidentical BE033 did not recognize cell lines infected with the Mariakani or buffalo-derived stabilates.

#### Immunized A18+ cattle vary in their CTL response to the Tp1 epitope

**Fig. 2:**
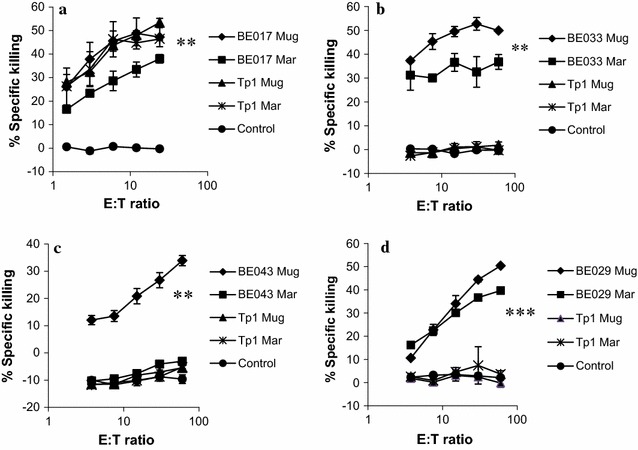
Peptide-specific CTL responses (cytotoxicity) in three A18+ cattle. BE017 (**a**), BE033 (**b**) and BE043 (**c**) and one control calf BE029 (**d**). CTL from the cattle were tested for killing on autologous cell lines infected with Muguga or Marikebuni (e.g. BE017-Mug or BE017-Mar) or autologous PBMC pulsed with the Tp1_214–224_ peptide from *T. parva* Muguga (Tp1 Mug) or *T. parva* Marikebuni (Tp1 Mar), as indicated. The experiment was performed twice with similar results. T-test was used for testing statistical significance. The highest E:T ratio was tested against the PBMC control. *P < 0.05, **P < 0.01, ***P < 0.001. Significance between killing of Muguga and Marikebuni was also tested and found to be significantly less (for Marikebuni) with at least P < 0.05 for the animals BE033, BE043 and BE029 but not for BE017

The pattern of killing of autologous infected cell lines suggested that animals sharing the A18 MHC haplotype differ in their CTL specificity, even when the animals are haploidentical. We examined this further by determining if there were differences in the CTL recognition of the Tp1_214–224_ epitope, previously shown to be presented by the A18 haplotype. This was assessed on two naturally occurring variants of the epitope, present in the *T. parva* Muguga and Marikebuni parasites, respectively, plus autologous cell lines infected with these parasite strains. There was a clearly different pattern of reactivity among the three A18+ animals (Fig. 2). BE017 appeared to recognize both variants of the Tp1_214–224_ epitope, plus the infected cell lines. Neither of the haploidentical animals BE033 and BE043 lysed the peptide-pulsed targets. We have previously shown that animals expressing either the allele BoLA-6*01301or BoLA-6*01302 recognize the Tp1_214–224_ epitope [26], so the lack of recognition of the peptide by BE033 and BE043 is not due to the variant A18 allele. Another difference was the lack of recognition by BE043 of the Marikebuni-infected cell line, which was well recognized by the haploidentical BE033 CTL. The results underscore the difference in the CTL specificity exhibited by haploidentical individuals.

**Fig. 3:**
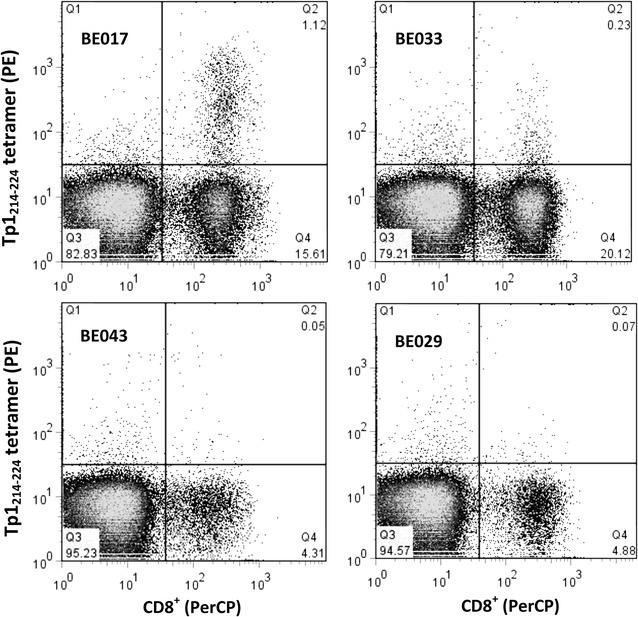
Muguga derived Tp1_214–224_ tetramer staining of PBMC from the three A18+ animals. BE017, BE033, BE043 and the control animal (BE029), isolated at day 15 after immunization. PBMC were co-stained with anti-CD8 Ab-PerCP and Tp1_214–224_-tetramer-PE. Initial gating was performed on the live lymphocyte fraction (not shown). Staining of the CD8^+^ cells by the tetramer is seen in the upper right quadrant (Q2). The animals from which the CD8^+^ cells were derived are indicated in the figure. The experiment was performed twice

Tetramer staining was performed on PBMC from the four animals at the optimal time point 15 days after challenge [27], and co-stained with Tp1_221–224_/BoLA 6*01301 tetramers and an anti-CD8 antibody. As shown in Fig. 3, cells from BE017 reacted very strongly with the tetramer, with 7.01% of CD8^+^ cells (or 1.12% of the total PBMC population) being positive. There appeared to be a very small percentage of positive cells in BE033 (0.23% of the total population), although when expressed as a percentage of the CD8^+^ cells (1.14%), there was no difference between BE033 and the control animal BE029 (1.43%). There was no difference between BE043 and the control animal. This experiment was repeated 6 months later with similar results but a lower level of tetramer positive cells in BE017 (data not shown). These results were corroborated by IFN-gamma ELISPOT assay (Additional file 2). Overall, these results support those observed in the cytotoxicity assays described above.

### Discussion

CTL are believed to be the major mechanism of protection using the Muguga cocktail and during natural immunity, which underscores the importance of analyzing the CTL response induced by the vaccine. Our aim in the current study was to test whether a mixture of parasite stabilates (Muguga cocktail) consistently induces CTL capable of killing cell lines infected with a broad array of parasite types. The results presented here suggest that this is not always the case.

CTL from BE017 killed all different infected cell lines, although there were differences in the efficiency of lysis. The BE017 CTL recognized the Tp1_214–224_ epitope, which is known to be relatively conserved among different *T. parva* strains, with only four different variant epitopes found in field samples thus far [28]. It is known that CTL cross-react with these variant epitopes [19] and the present results confirm this observation.

Surprisingly, the CTL response in both BE033 and BE043 showed no reactivity to the Tp1_214–224_ epitope. This may be due to the other MHC haplotype (A12) since it has been shown that the CTL response can be dominated by one haplotype [29].

While BE043 efficiently lysed all *T. parva* strains tested, except *T. parva* Marikebuni, BE033 showed a variable response among the different strains and BE043 didn’t kill the Marikebuni strain, so the CTL in this calf must be dominated by a specificity that is not cross-reactive with the Marikebuni strain, presumably through the presentation of a non-crossreactive epitope. This difference between Muguga and Marikebuni has been studied previously, mostly in context of strain-specific clones [13, 30]. While this is interesting from an immunological point of view, it is the overall polyclonal response which is more important from a vaccine perspective because this will inform if the immune response has the potential to cross-protect between strains. Unfortunately, there are few of these studies which would provide valuable information for improvement of a live vaccine. The Muguga and the Marikebuni strains have obviously some differences and a Marikebuni strain could be considered for inclusion in a live vaccine.

BE033 had the most variable CTL response of the three animals with regards to strain specificity. Such variability may potentially lead to breakthrough in the field, i.e. incidences of ECF despite vaccination, for certain combinations of host MHC haplotypes and infection with some *T. parva* strains.

In summary, these findings suggest that the specificity of the CTL response following immunization with a live vaccine against *T. parva* varies among animals sharing the same MHC haplotype, including a pair of haploidentical individuals, and that certain strain/MHC haplotype combinations could possibly lead to break through of the vaccine. Further, the variable immune responses found in MHC class I haploidentical cattle most likely translate to other species as the mechanisms in generation of CTL responses are very similar across species.

### Limitations

The animal number is small. It would be preferable to have more haploidentical animals in the study.

## Additional files

**Additional file 1.** Table showing sequence-specific primer sequences used for BoLA Class I typing.

**Additional file 2.** IFN-γ release from CD8^+^ cells derived from the three A18+ cattle. BE017, BE033 and BE043 and one control calf (BE029) in response to (A) autologous Muguga 4230 infected cell lines and (B) Tp1_214–224_ peptide derived from Muguga. The relative numbers of spots compared to added cells are shown (frequency). A positive control cell line was included (CTL line) which reached higher values than the maximum on the Y-axis. The maximum was diminished to visualize the important results. Statistical significance was tested with a *T* test, (*) P < 0.05. (**) P < 0.01.

## Abbreviations

CTL: cytotoxic T-lymphocyte
ECF: East Coast fever
MHC: major histocompatibility complex
ITM: infection and treatment method
BoLA: bovine leucocyte antigens
PBMC: peripheral bovine mononuclear cells
IFN: interferon
