# Supplementary material for: Cytotoxic T lymphocytes from cattle sharing the same MHC class I haplotype and immunized with live Theileria parva sporozoites differ in antigenic specificity
Source: BMC Res Notes. 2018 Jan 17;11:44. doi: 10.1186/s13104-018-3145-8 (PMC5773172; doi:10.1186/s13104-018-3145-8)
Supplement: Supplementary file 1 — Additional file 1. Table showing sequence-specific primer sequences used for BoLA Class I typing. [file 13104_2018_3145_MOESM1_ESM.docx]

| **Haplotype** | **Forward primer sequence** | **Reverse primer sequence** |
| --- | --- | --- |
| A10 | 5’-CTC CCA CTC GAT GAG GTA T-3’ | 5’-ATC TGA GCC ATC GTC TCC A-3’ |
| A11 | 5-’CAC GCA GTT CAC ACG GTT-3’ | 5’-CGA ACT GCA TGA ACC CGA-3’ |
| A12 | 5’-CCC GCT TCA TCA CCG TT-3’ | 5’-TCG TAG CCG TAC TGA TCA-3’ |
| A14 | 5’-CCG TGG ATA GAG AAG GAA-3’ | 5’-CAA AGA CTC AGC ATA ACC TT-3’ |
| A15 | 5’-ATG GAG CCG CGG GCG CCG TGG ATA-3’ | 5’-AAA GAC TCA GCA TAA CCT TCC-3’ |
| A17 | 5’-GAG GTA TTT CTA CAC CGG-3’ | 5’-TCG TAG CCG AAC TGC GTA-3’ |
| A18 | 5’-CCG GGA TCC GAG GAC T-3’ | 5’-CTC CAT CTT GCG TTT GGA-3’ |
| A19 | 5’-CCG GGA GCC CCT CTT T-3’ | 5’-GCC GTA CAT CCG CTG AT-3’ |
| A20 | 5’-CAG ATT ATG CTG AGT CTT TG-3’ | 5’-CAG ATT ATG CTG AGT CTT TG-3’ |
| A31 | 5’-GAG CCG CGC TTC ATC TCT-3’ | 5’-GAG CCG CGC TTC ATC TCT-3’ |

Additional file 1

**Table 1**. Sequences of sequence-specific primers (SSP) used for BoLA class I typing.
