# Supplementary material for: Cytotoxic T lymphocytes from cattle sharing the same MHC class I haplotype and immunized with live Theileria parva sporozoites differ in antigenic specificity
Source: BMC Res Notes. 2018 Jan 17;11:44. doi: 10.1186/s13104-018-3145-8 (PMC5773172; doi:10.1186/s13104-018-3145-8)
Supplement: Supplementary file 2 — Additional file 2. IFN-γ release from CD8+ cells derived from the three A18+ cattle. BE017, BE033 and BE043 and one control calf (BE029) in response to (A) autologous Muguga 4230 infected cell lines and (B) Tp1214–224 peptide derived from Muguga. The relative numbers of spots compared to added cells are shown (frequency). A positive control cell line was included (CTL line) which reached higher values than the maximum on the Y-axis. The maximum was diminished to visualize the important results. Statistical significance was tested with a T test, (*) P < 0.05. (**) P < 0.01. [file 13104_2018_3145_MOESM2_ESM.docx]

**A**

**B**

**

*

**

**

**
